# Sarcoma of Tonsil—Cancer of Breast—Clinic at Buffalo General Hospital

**Published:** 1895-02

**Authors:** Roswell Park

**Affiliations:** Professor of Surgery, University of Buffalo


### SARCOMA OF TONSIL—CANCER OF BREAST—CLINIC AT BUFFALO GENERAL HOSPITAL.

By ROSWELL PARK, A. M., M. D.,
Professor of Surgery, University of Buffalo.

### I.—SARCOMA OF THE TONSIL.

Here is a man who presents a hard enlargement on the side of the neck. The skin is reddened over it, but this is due to the recent application of a poultice. It is well to distinguish between an artificial redness of this sort and inflammatory rubor. If it were inflammatory, I should expect the skin to be less freely movable. The tumor is in and around the sterno-cleido-mastoid muscle and quite firmly blended with it. It is very firm and hard and only slightly movable. It does not roll around beneath the skin, but has pretty firm, deep adhesions. It reaches to the jaw and is about the size of a hen's egg.

I will next examine for any enlarged lymph-nodes that may be present. I can feel none. The patient says that he has had the growth "all Summer," and this is as definite information as we can obtain. His speech is thick and his voice hoarse. This is not his normal voice, and the patient tells me that he feels as if there were an enlargement of his throat. Internally, there is a large irregular fungous mass, filling up half the pharynx. The mass is jagged and more or less ulcerated, deserving the name of fungous growth. The diagnosis is sarcoma. This diagnosis could have been made without looking in the throat, but the view that I got made it easy. Whether it began in the tonsil or outside, there is now no means of knowing. Primary sarcoma of the tonsil is rare, there being not more than thirty or forty cases on record. The case is necessarily fatal, and speedily so, for such growths enlarge in arithmetical progression. It will soon become difficult for the patient to swallow, and later, even breathing will become difficult. I should not advise operation in this case, for it would be terribly severe, and the trouble would soon return. I have had one case of primary sarcoma of the tonsil, in which I split the jaw and took out all that side of the throat. The patient is now, two years after the operation, perfectly well. In clinic, a short time ago, I did a similar operation, and the patient died soon after. In this I do not think a total extirpation would be practicable, and unless the whole tumor is removed, there can be no hope of saving or materially prolonging life. In the last case of this kind, to which I have just referred, the patient was threatened with suffocation, and, with death staring him in the face, he begged to have the chance of relief, at any risk. I should be averse to operating under the present circumstances. Later, a tracheotomy may be indicated. Or, if the pressure is more on the esophagus and the man is in danger of starving to death, a gastrostomy might be done, in order to allow artificial feeding. But there is no averting the doom, and I shall not think of operating except for temporary relief.

### II.—CANCER OF THE BREAST.

The first operative case this morning is a woman, 51 years of age, who has foolishly allowed a large tumor of the breast to go on for a year, under the delusion that it might be helped by medicine, in which hope, I am sorry to say, she has been encouraged by a man who is practising medicine. I will very briefly run over the indications of its malignant character and will then remove the breast. The breast itself is still somewhat movable over the deep fascia. If there were firm adhesions to the pectoral fascia, I should hesitate about attacking the tumor at all, thinking that she would live longer without operation than with it. In this case, however, we hope to prolong life, if not to save it. Twice within the last few years I have seen malignant disease in this location lighted up into renewed and awful activity by untimely interference. Still, if operation were refused in all cases in which the hope of absolute cure could not be entertained, great injustice would be done to many sufferers from malignant disease. The patient has a fleshy axilla, and from the outside I do not detect any lymphatic enlargement, but it is not safe to consider her free from such metastasis. The lump is hard and there is no reasonable doubt that the tumor is a scirrhous cancer. The entire mammary gland must be sacrificed and a good deal of surrounding tissue for fear of recurrence. Ordinarily, the incision is made elliptically with the long axis pointing downward and inward, but I shall reverse the usual incision and make a broader ellipse with the longer axis pointing downward and outward. In one place you can see that the growth has gone nearly to the pectoral fascia, and I shall deliberately remove most of the pectoralis major. The sacrifice of muscle is a minor point in comparison with the importance of removing every vestige of the neoplasm. Now, extending the incision toward the axilla, you see, I come at once upon an enlarged lymph-node. This indicates that the axilla must be cleaned out—indeed it is a safe rule to do so in any operation for malignant tumor of the breast.

The operation in the axilla will occupy more time than the original removal of the breast. It is necessary to expose the axillary vein, and the man who hesitates to do this had better not undertake to remove a breast. One or two small branches of the axillary vein are broken through in clearing away the connective tissue and fat from the lymph-nodes which lie deeper. These veins I will catch now with hemostats and will ligate later. Last year, while doing an operation for recurrent cancer, in the course of which I had to go higher in the axilla than is here necessary, I tore off a large branch of the axillary vein so close to the main vessel that ligation was impossible. I knew that if I ligated the axillary vein there would be established a permanent edema of the arm, and I therefore adopted a method of procedure which is coming into vogue with a few surgeons, and closed the tear in the wall of the vein with a couple of hemostats, suturing the wound with the forceps in place. After four days they were removed and not a drop of secondary hemorrhage followed.

In the present case, after clearing out the axilla as completely as possible, I have only two or three lymph-nodes and a few tags of fat to remove. The wound is closed with buried sutures and the ordinary cutaneous suture of catgut. There has been, throughout the operation, much less hemorrhage than usual. I have taken the ground that drainage, in any fresh, aseptic case, is a confession of ignorance or of fear. In the present case, I shall leave a small outlet for drainage through an absorbable bone tube which will disappear in a few days. I do so because I have some fear that part of the tissues left may break down and slough away. After the insertion of the drainage tube and the completion of the sutures, I make pressure in order to force out whatever serum and blood has been left in the wound—namely, to dry it. I feel now as confident of good result as one ever can in any operation for similar disease. The field of operation is smeared with sterilized and antiseptic vaseline to prevent the sticking of the dressings, powdered with iodoform, and the wound is covered with strips of iodoform gauze, over which is laid the ordinary dressing of bichlorid gauze and cotton.

Here is the tumor split in two, and one side I show you in the normal condition, while the other has been subjected to Stile's test, which consists in the application of a 10 per cent. watery solution of nitric acid. This coagulates the albumin of the tumor and gives it a cartilaginous appearance, the dense whiteness of the tumor showing in bold relief against the yellowish, fatty tissue surrounding it. In this way, the test of the thoroughness of the operation can be made by an assistant while the vessels are being ligated, and if any ramification of the tumor is seen going up to the edge of the tissue removed, a further excision can be made at that point.

